# Association between all-cause mortality and trajectories across quality and duration of sleep and cognitive function: based on Group-Based Multivariate Trajectory modeling

**DOI:** 10.1186/s12877-023-04231-3

**Published:** 2023-08-30

**Authors:** Jianlin Lin, Jian Xiao, Qiao Li, Li Cao

**Affiliations:** 1 International School of Public Health and One Health, Hainan Medical University, No. 3 Xueyuan Road, Longhua District, Haikou, Hainan 571199 China; 2 Department of Oral and Maxillofacial Surgery, Stomatological Center, Peking University Shenzhen Hospital, Shenzhen, China; 3 Guangdong Provincial High-Level Clinical Key Specialty, Shenzhen, China; 4 Guangdong Province Engineering Research Center of Oral Disease Diagnosis and Treatment, Shenzhen, China; 5 Nanjing Municipal Center for Disease Control and Prevention, Nanjing, 210003 China

**Keywords:** Older adults, Sleep quality, Sleep duration, Cognitive function, Trajectory

## Abstract

**Background:**

Sleep duration and quality are associated with cognition, but the interaction of the 3 indicators and their association with all-cause mortality is unclear.

**Methods:**

We used data from the Chinese Longitudinal Healthy Longevity Survey from 2005–2018 to identify latent trajectories of sleep duration, sleep quality, and cognitive function. Secondly, the multinomial logistic model was adopted to determine predictors of trajectory groups. Finally, the Cox regression model was used to examine the association between these trajectory groups and all-cause mortality.

**Results:**

A total of 5046 adults (49% women) with an average age of 76.34 were included in the study. The median follow-up period was 11.11 years, during which 1784 (35%) participants died. We identified 4 latent groups among older adults: ‘Good-performance’ (51%), ‘Decreasing’ (26%), ‘Oversleep & cognitive impairment’ (12%), and ‘Sleep-deprived’ (11%). Individuals in the ‘Decreasing’ had a 51% increased risk of all-cause mortality (HR = 1.51, 95% CI: 1.25 – 1.81, *p* < .001). Individuals in the ‘Oversleep & cognitive impairment’ had a 170% increased risk of all-cause mortality (HR = 2.7 95% CI: 2.13 – 3.43, *p* < .001). Women had a higher risk of all-cause mortality regardless of trajectory group (47–143% men VS. 74–365% women). Both urban and rural areas have a similarly increased risk of all-cause mortality (48–179%).

**Conclusions:**

Our study reveals the latent trajectories across sleep duration, sleep quality, and cognitive function in older Chinese and further explores their association with death. These findings provide a rational basis for cognitive interventions and reduce all-cause mortality.

**Supplementary Information:**

The online version contains supplementary material available at 10.1186/s12877-023-04231-3.

## Introduction

As aging in Chinese develops by leaps and bounds, the burden of all-cause mortality of older adults in China has significantly grown. Extensive studies have shown that sleep quality, sleep duration, and cognitive impairment are associated with all-cause mortality in older adults. However, these findings are often very fragmented. First, the interrelationship between sleep quality, sleep duration, and cognitive function is complex and not well understood. Second, the effect of these interrelationships on death has not been quantified.

In recent research, the association between sleep problems (duration and quality) and cognitive function has gradually attracted attention. But their associations are knotty and not well explored. On the one hand, some studies have identified sleep problems as a curative factor [1]. Cumulative studies have shown that moderate sleep duration is a positive factor for maintaining optimal cognitive function and oversleeping or shorter sleep duration may lead to cognitive decline. However, these findings may be contradictory. For example, some studies have found a U-shaped relationship between sleep duration and cognitive impairment, that is, less than 6 h or more than 9 h may increase the risk of cognitive impairment [2, 3]. In some studies, only excessive sleep duration was associated with cognitive impairment [4, 5].

On the other hand, changes in sleep duration may cause by modifications in cognitive function over time. Recent evidence suggests that individuals with cognitive decline were more likely to get excessive sleep [6]. However, some researchers have found that moderately increased sleep duration is not related to cognitive decline, but is associated with an increase in overall cognitive scores [7]. Other studies have shown that participants with 6–8 h sleep duration shifted to higher or lower is associated with cognitive impairment [8, 9]. Although studies have examined sleep duration and cognitive trajectories, they have ignored sleep quality [10]. Along with sleep duration, sleep quality is also significantly associated with cognitive function [11, 12]. Extensive research has shown that poor sleep quality is associated with cognitive impairment, including memory and processing capacity in older adults [12, 13].

However, most previous studies that investigated the association between sleep disorder and cognitive function have had significant limitations. First, findings from cross-sectional studies and analysis in sequence are not supported to examine the association between sleep quality and cognitive function. Thus, understanding the potential combination of sleep duration and quality in cognitive function has important public health practical implications for the prevention of cognitive impairment. Second, cognitive impairment is typically defined as an outcome variable in the final wave. The absence of cognitive function in baseline may not rule out reverse causation, as participants with poor cognitive function are more likely to have sleep problems [14]. Third, although studies have considered the association between sleep duration, sleep quality, and cognition, follow-up is too short. Yet a short follow-up did not enable us to assess the association between trajectory in sleep quality and cognitive function. More importantly, the changes in sleep duration and sleep quality were not observed in conjunction with the cognitive trajectories. It is not clear whether there is a covariant relationship between sleep duration, sleep quality, and cognitive function, and the health significance or connotation of its existence.

Aside from the complex association between sleep problems and cognition, the relationship between these associations and death is unclear. Previous studies have been fraught with inconsistencies. For example, short sleep duration and poor sleep quality are often risk factors for mortality. Some studies suggest that the interaction between sleep quality and duration is not associated with mortality, while another study showed that poor sleep duration and quality increased the risk of cardiovascular and cerebrovascular death (increased by 63%) for participants with unhealthy baselines. In addition, studies with shorter follow-ups (less than 10 years) were unable to investigate the significance of the health of older adults in the association between all-cause mortality and 3 indicator trajectories over time (sleep duration, sleep quality, and cognitive function).

In short, previous studies have some limitations. In terms of study design, mixed results were obtained from shorter observation times, smaller sample sizes, and baseline data that did not include cognitive function.In terms of research content, on the one hand, previous studies only focused on the independent effects of sleep time and sleep quality on cognition, and rarely examine the covariant effect of the 3 indicators (sleep duration, sleep quality, and cognitive function) over time. On the other hand, the significance of these covariant trajectories (sleep duration, sleep quality, and cognitive function) for all-cause mortality is unknown. To fill in this gap, this work aimed to 1) explore the optimal trajectory shape of sleep duration, sleep quality, and cognitive function together over time by using nationally representative data on Chinese older adults from 2005 to 2018; 2) explore the predictive factors of multivariate trajectories, and 3) explore the association between the change of 3 indicators over time and all-cause mortality.

## Methods

### Data source

The data were obtained from the Chinese Longitudinal Healthy Longevity Survey (CLHLS), which surveyed in 2005, 2008, 2011, 2014, and 2018. This is the largest and nationally representative survey of older adults in China, covering 22 cities and counties in 31 provinces of China. The CLHLS has oversampled the older adults, with weighted by age, sex, and rural-urban. Detailed information about the calculation of sample size was presented in the additional file (Supplemental Data source, Additional file 1). The questionnaire was conducted by professionally trained personnel with face-to-face interviews and controlled by quality controllers. Detailed study design information can be found in previous studies [15]. We included a total of 5046 participants aged 60 years old or above and participated in at least 3 waves including full details about sleep quality, sleep duration, and cognitive function.

### Measures

#### Measures of trajectory

The trajectory modeling was based on 3 indicators: sleep quality, sleep duration, and cognitive function. Sleep quality was measured by the question: ‘How do you rate your recent sleep quality?’ (1 = very bad, 2 = bad, 3 = fair, 4 = good, or 5 = very good) [16]. by the number of hours spent sleeping normally. Cognitive function was evaluated by the Chinese version of the mini-mental state examination (MMSE; 0–30 scores), with a standardized Cronbach’s α of 0.87. Lower scores indicate more severe cognitive impairment [17–19].

#### All-cause of mortality

The outcome was measured by all-cause of mortality. Participants' vital status and date of death were determined through interviews with family members in each follow-up survey. The quality of mortality data was verified based on previous assessments [20].

#### Covariates

Covariates include age, gender (men, women), marital status (in marriage, not in marriage), residence (urban, rural), education (illiterate, literate or primary school, and junior high and above), occupation (low level, high level), regular exercise (no, yes), smoke (no, yes), drink (no, yes), disability, and the number of chronic diseases (0, 1–2, > 2). All covariates were extracted with baseline. Disability was measured by the Chinese version of the Katz’s activities of daily living (ADL) scale (standardized Cronbach’s α = 0.91) [21, 22]. Disability was defined as needing any assistance in performing the 6 activities: bathing, dressing, toilet, indoor transfer, continence, and eating. Questions about these covariates and how to classify them can be found in the additional file (Supplemental Measures, Additional file 1).

### Statistical analyses

Baseline characteristics, grouped by vital status, were compared by a t-test for continuous variables and χ^2^-test categorical variables. The discrete variables are presented as numbers (percentage), and the continuous variables are presented as mean (SD) or median (interquartile range [IQR]). First, Group-Based Multivariate Trajectory modeling (GBMT) was a longitudinal data modeling method based on multiple continuous indicators [23], which enables the estimation of missing values using the Expectation-Maximization algorithm. GBTM in this work was used to explore latent groups of participants following similar trajectories based on sleep quality, sleep duration, and cognitive function. To determine the trajectory’s shape, the polynomial degree of group trajectories was set as 2. Model selection was based on statistical indicators (the smallest Bayesian Information Criterion [BIC], Akaike information criterion [AIC], Consistent AIC [CAIC], Hannan-Quinn information criterion [HQIC], Sample size-adjusted Bayesian information criterion [ssBIC]) [24–26] and explanatory and meaningful over multiple interesting dimensions (having at least 5% of subjects in a group) [27]. If the trajectory’s shape is similar in different groups, it is reasonable to combine these trajectories considering the interpretability and simplicity of the results. Second, a multinomial logistic model was performed to assess the factors affecting the multivariate trajectory group. Third, Cox regression was conducted to assess the association between all-cause mortality and the latent groups of trajectory, adjusting for covariates. Data management is implemented using R (version 4.2.2). GBMT was conducted in the "gbmt" package in R [28]. *P* < 0.05 was considered statistically significant.

## Results

### Sample characteristics

Table 1 shows the baseline characteristics of older adults. The study included 5,046 participants (49 percent women), with 1784 (35%) participants dead during follow-up. The mean age was 76.34 ± 9.28 years and the median follow-up duration was 11.11 [IQR, 9.44–14.11] years). Significant differences were revealed between survival or censor and death in age, education, occupation, residence area, current marital status, regular exercise, and disability.

**Table 1:** Basic characteristics for Chinese older adults aged 60 years or above

	**Total**	**Survical or censor**	**Death**	***P*-value**
**Age**				
Mean (SD)	76.34 (± 9.28)	73.55 (± 7.98)	81.36 (± 9.33)	< 0.001
**Gender**				
Women	2,473 (49.01)	1,616 (49.54)	857 (48.04)	0.32
Men	2,573 (50.99)	1,646 (50.46)	927 (51.96)	
**Education**				
Illiterate	178 (6.70)	108 (5.78)	70 (8.88)	< 0.001
Literate or primary school	1,826 (68.75)	1,240 (66.38)	586 (74.37)	
Junior high or above	652 (24.55)	520 (27.84)	132 (16.75)	
**Occupation**				
Low level	4,500 (89.45)	2,880 (88.56)	1,620 (91.06)	0.006
High level	531 (10.55)	372 (11.44)	159 (8.94)	
**Residence area**				
Rural	3,211 (63.63)	2,048 (62.78)	1,163 (65.19)	0.092
City	1,835 (36.37)	1,214 (37.22)	621 (34.81)	
**Current marital status**				
No	2,099 (41.61)	1,117 (34.25)	982 (55.04)	< 0.001
Yes	2,946 (58.39)	2,144 (65.75)	802 (44.96)	
**Regular exercise**				
No	3,236 (64.16)	2,042 (62.64)	1,194 (66.93)	0.002
Yes	1,808 (35.84)	1,218 (37.36)	590 (33.07)	
**Drinking**				
No	3,767 (74.68)	2,420 (74.21)	1,347 (75.55)	0.31
Yes	1,277 (25.32)	841 (25.79)	436 (24.45)	
**Smoking**				
No	1,341 (26.58)	867 (26.58)	474 (26.58)	1
Yes	3,704 (73.42)	2,395 (73.42)	1,309 (73.42)	
**Disability**				
No	4,824 (95.70)	3,172 (97.39)	1,652 (92.60)	< 0.001
Yes	217 (4.30)	85 (2.61)	132 (7.40)	
**Number of chronic**				
0	1,493 (29.59)	983 (30.13)	510 (28.59)	0.1
1–2	3,021 (59.87)	1,956 (59.96)	1,065 (59.70)	
> 2	532 (10.54)	323 (9.90)	209 (11.72)	
**Sleep quality**				
Median [IQR]	4.00 [3.00,4.00]	4.00 [3.00,4.00]	4.00 [3.00,4.00]	0.332
**Sleep duration**				
Median [IQR]	8.00 [6.00,9.00]	8.00 [6.00,9.00]	8.00 [6.00,8.00]	< 0.001
**MMSE**				
Median [IQR]	29.0 [26.0,30.0]	28.0 [25.0,29.0]	29.0 [27.0,30.0]	< 0.001

### Model select and affecting factors on trajectories

**Fig. 1:**
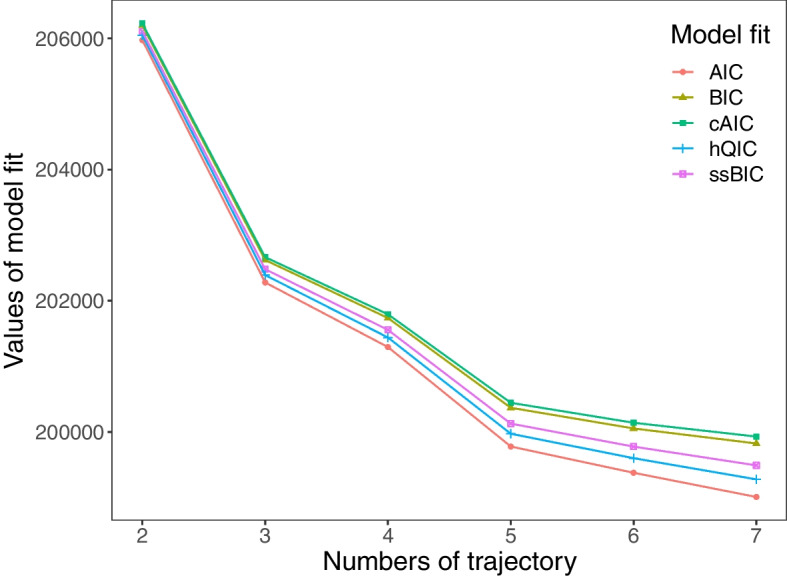
Statistical metrics for model selection

**Fig. 2:**
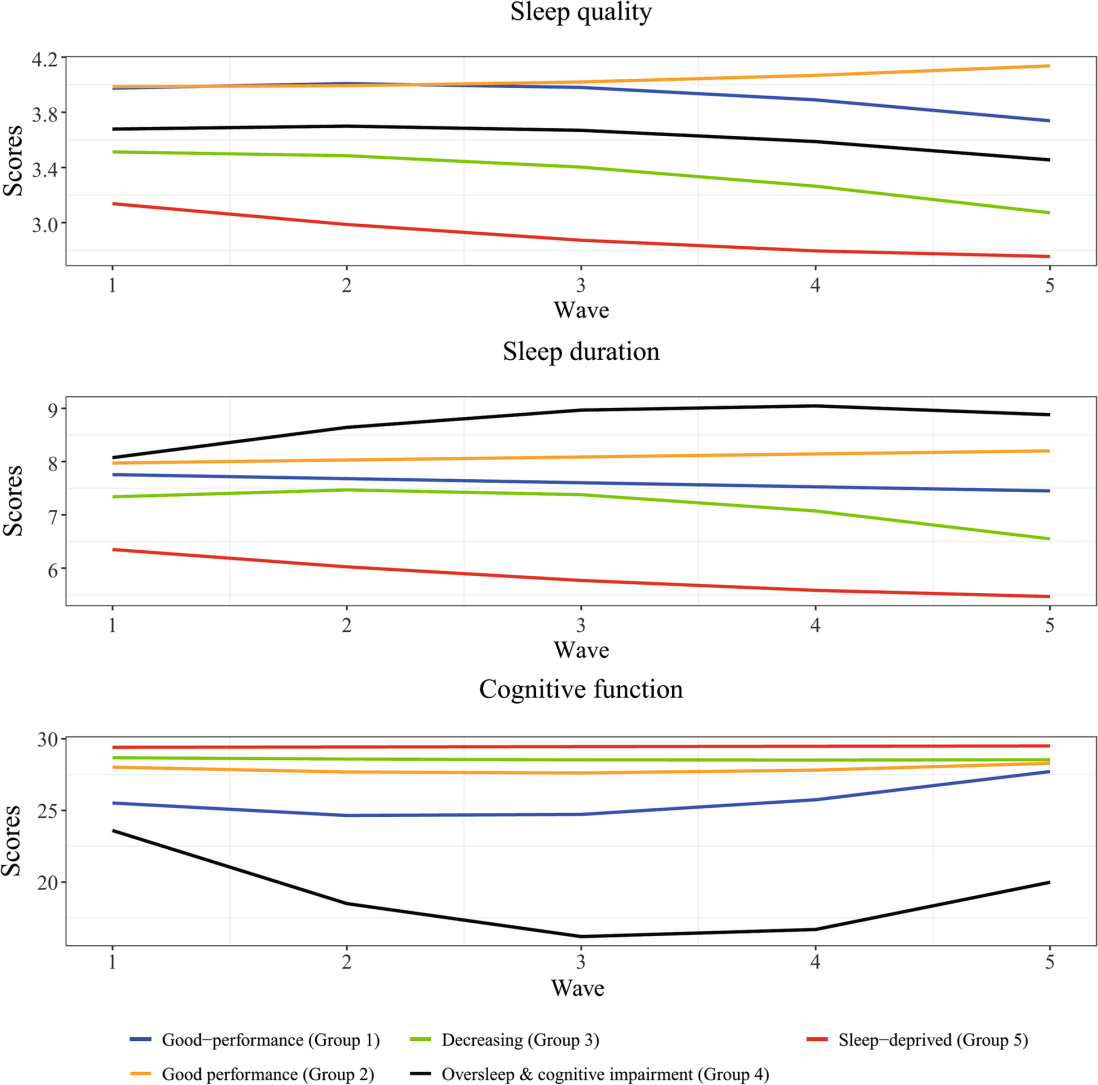
Latent trajectory shape for 5 groups across sleep quality, sleep duration, and cognition function

Model selection was based on interpretation and statistical indicators. Figure 1 shows that the value of the statistical indicator decreases with the increase of groups. After the fifth group’s slope becomes significantly flattened, thus, the 5 groups were available. Figure 2 presented the different trends of 5 groups. Group 1 (22%) and group 2 (29%) seemed consistent to maintain a high level of cognitive function with quality sleep and average sleep duration. Thus, they were described as ‘Good-performance’ and merged into a common group in subsequent analysis. The participants in group 3 were more probably to have decreased quality and duration of sleep and average cognition. Thus, group 3 was tagged as ‘Decreasing’ (26%). The participants in group 5 were more apparent with worst sleep quality and duration, and cognition. Thus, group 4 was labeled as ‘Oversleep & cognitive impairment’ (12%). The participants in group 5 were more seemingly to have secondary cognition and worse quality and duration of sleep. Thus, group 5 was interpreted as ‘Sleep-deprived’ (11%).

Factors associated with the latent trajectories were measured by multinomial logistic regression (Table 2). Older participants, lower education, and living in rural were more likely to belong to the ‘Decreasing’ group, compared with the group of ‘Good-performance’. Men were more likely to belong to the ‘Sleep-deprived’ group. Older participants with lower education were more likely to belong to the ‘Oversleep & cognitive impairment’ group.

**Table 2:** Predictor for the latent trajectories across sleep duration, sleep quality, and cognitive function

	**Decreasing**	**Oversleep & cognitive impairment**	**Sleep-deprived**
**Gender**			
Women			
Men	0.54 (0.40–0.73, *p* < .001)	0.78 (0.47–1.28, *p* = .326)	0.57 (0.43–0.77, *p* < .001)
**Age**			
Mean ± SD	1.09 (1.07–1.10, *p* < .001)	1.14 (1.12–1.17, *p* < .001)	0.99 (0.98–1.01, *p* = .469)
**Education**			
Illiterate			
Literate or primary school	0.47 (0.32–0.69, *p* < .001)	0.58 (0.30–1.12, *p* = .103)	0.93 (0.55–1.55, *p* = .770)
Junior high or above	0.30 (0.19–0.50, *p* < .001)	0.33 (0.15–0.75, *p* = .008)	0.82 (0.47–1.45, *p* = .501)
**Occupation**			
Low level			
High level	0.76 (0.51–1.12, *p* = .166)	0.93 (0.52–1.66, *p* = .801)	1.31 (0.94–1.82, *p* = .110)
**Residence area**			
Rural			
City	0.70 (0.54–0.92, *p* = .009)	0.93 (0.62–1.41, *p* = .738)	1.06 (0.82–1.38, *p* = .639)
**Current marital status**			
No			
Yes	0.78 (0.60–1.01, *p* = .059)	0.67 (0.44–1.00, *p* = .051)	1.15 (0.86–1.53, *p* = .345)
**Regular exercise**			
No			
Yes	0.76 (0.59–0.98, *p* = .036)	0.99 (0.66–1.48, *p* = .964)	0.98 (0.76–1.26, *p* = .857)
**Drinking**			
No			
Yes	0.95 (0.72–1.25, *p* = .715)	1.30 (0.83–2.04, *p* = .245)	0.94 (0.71–1.23, *p* = .641)
**Smoking**			
No			
Yes	0.89 (0.68–1.18, *p* = .422)	0.99 (0.63–1.57, *p* = .969)	0.95 (0.72–1.25, *p* = .709)
**Disability**			
No			
Yes	2.18 (1.20–3.97, *p* = .010)	4.42 (2.21–8.82, p < .001)	0.44 (0.13–1.47, *p* = .184)
**Number of chronic**			
1			
1–2	1.01 (0.78–1.32, *p* = .918)	1.47 (0.93–2.32, *p* = .100)	0.87 (0.67–1.12, *p* = .270)
> 2	1.29 (0.86–1.95, *p* = .222)	1.84 (0.95–3.58, *p* = .072)	0.71 (0.45–1.12, *p* = .137)

### Associations between all-cause mortality and trajectories

Based on the Cox regression model, we investigated the association between latent trajectory groups and all-cause mortality (Table 3). Compared to the ‘Good-performance’ group, individuals in the ‘Decreasing’ had a 51% increased risk of all-cause mortality (hazard ratio [HR] = 1.51, 95% CI: 1.25 – 1.81, *p* < 0.001). Individuals in the ‘Oversleep & cognitive impairment’ had a 170% increased risk of all-cause mortality (HR = 2.7 95% CI: 2.13 – 3.43, *p* < 0.001).

**Table 3:** The association between the latent trajectories and all-cause mortality based on Cox regression

	**All**	**Men**	**Women**	**Rural**	**City**
**Group**					
Good-performance					
Decreasing	1.51 (1.25–1.81, *p* < .001)	1.47 (1.20–1.82, *p* < .001)	1.74 (1.14–2.65, *p* = .010)	1.48 (1.18–1.86, *p* = .001)	1.53 (1.12–2.09, *p* = .007)
Oversleep & cognitive impairment	2.70 (2.13–3.43, *p* < .001)	2.43 (1.85–3.20, *p* < .001)	4.65 (2.78–7.80, *p* < .001)	2.79 (2.02–3.85, *p* < .001)	2.62 (1.82–3.77, *p* < .001)
Sleep-deprived	1.10 (0.87–1.39, *p* = .433)	1.03 (0.78–1.34, *p* = .846)	1.61 (0.97–2.66, *p* = .064)	0.92 (0.66–1.27, *p* = .601)	1.42 (1.01–2.00, *p* = .044)
**Gender**					
Women					
Men	1.76 (1.45–2.14, *p* < .001)			1.61 (1.23–2.10, *p* < .001)	1.94 (1.45–2.61, *p* < .001)
**Age**					
Mean ± SD	1.06 (1.05–1.07, *p* < .001)	1.05 (1.04–1.06, *p* < .001)	1.07 (1.05–1.09, *p* < .001)	1.07 (1.05–1.08, *p* < .001)	1.05 (1.03–1.06, *p* < .001)
**Education**					
Illiterate					
Literate or primary school	0.95 (0.74–1.23, *p* = .719)	0.96 (0.71–1.31, *p* = .807)	0.99 (0.62–1.59, *p* = .976)	1.07 (0.79–1.45, *p* = .661)	0.75 (0.47–1.21, *p* = .244)
Junior high or above	0.65 (0.47–0.88, *p* = .006)	0.66 (0.46–0.94, *p* = .022)	0.45 (0.21–0.95, *p* = .037)	0.81 (0.54–1.21, *p* = .312)	0.44 (0.26–0.74, *p* = .002)
**Occupation**					
Low level					
High level	1.08 (0.88–1.33, *p* = .451)	1.03 (0.82–1.28, *p* = .797)	1.61 (0.93–2.78, *p* = .089)	1.01 (0.73–1.40, *p* = .949)	1.11 (0.85–1.45, *p* = .449)
**Residence area**					
Rural					
City	0.81 (0.69–0.95, *p* = .010)	0.83 (0.70–0.99, *p* = .041)	0.68 (0.47–0.99, *p* = .042)		
**Current marital status**					
No					
Yes	0.81 (0.69–0.95, *p* = .009)	0.84 (0.71–1.01, *p* = .060)	0.64 (0.45–0.92, *p* = .017)	0.82 (0.67–1.00, *p* = .051)	0.80 (0.62–1.04, *p* = .094)
**Regular exercise**					
No					
Yes	0.86 (0.74–1.00, *p* = .057)	0.88 (0.75–1.05, *p* = .157)	0.86 (0.61–1.22, *p* = .406)	0.90 (0.73–1.10, *p* = .296)	0.79 (0.63–1.00, *p* = .052)
**Drinking**					
No					
Yes	0.84 (0.72–0.99, *p* = .036)	0.86 (0.72–1.01, *p* = .068)	0.65 (0.35–1.20, *p* = .168)	0.82 (0.67–1.00, *p* = .054)	0.88 (0.67–1.15, *p* = .354)
**Smoking**					
No					
Yes	0.93 (0.80–1.09, *p* = .395)	0.95 (0.80–1.12, *p* = .531)	0.98 (0.54–1.80, *p* = .959)	0.87 (0.71–1.07, *p* = .188)	1.05 (0.80–1.37, *p* = .729)
**Disability**					
No					
Yes	0.94 (0.69–1.30, *p* = .722)	0.86 (0.59–1.26, *p* = .438)	1.51 (0.81–2.82, *p* = .189)	0.87 (0.55–1.38, *p* = .560)	1.04 (0.66–1.64, *p* = .863)
**Number of chronic**					
1					
1–2	1.18 (1.01–1.39, *p* = .042)	1.21 (1.01–1.45, *p* = .041)	1.16 (0.80–1.68, *p* = .431)	1.16 (0.94–1.44, *p* = .167)	1.20 (0.93–1.55, *p* = .168)
> 2	1.05 (0.81–1.36, *p* = .728)	1.07 (0.80–1.43, *p* = .669)	0.97 (0.54–1.74, *p* = .909)	1.12 (0.80–1.56, *p* = .526)	0.98 (0.65–1.48, *p* = .925)

### Associations between all-cause mortality and trajectories grouped by gender, residence, and age

Compared to the ‘Good-performance’ group, participants in both groups ‘Decreasing’ and ‘Oversleep & cognitive impairment’ had a higher risk of all-cause mortality for both men and women, and women had a higher risk of death than men in these groups (men: HR = 1.47; HR = 2.43, *P* < 0.05, respectively; women: HR = 1.74; HR = 4.65, *P* < 0.05, respectively). Participants in both groups ‘Decreasing’ and ‘Oversleep & cognitive impairment’ had a higher risk of all-cause mortality for both living in rural and city (rural: HR = 1.48; HR = 2.79, *P* < 0.05, respectively; city: HR = 1.53; HR = 2.62, P < 0.05, respectively). While participants of ‘Sleep-deprived’ group who lived in the city were associated with a higher risk of all-cause mortality (HR = 1.42, *P* < 0.05). Younger individuals were more likely to have higher education (Supplemental Table 1, Additional file 1). In our analysis grouped by age, there is still a stable association between all-cause mortality and trajectories, with the strongest associations in both ‘Decreasing’ and ‘Oversleep & cognitive impairment’ groups (Supplemental Table 2, Additional file 1).

## Discussion

Based on 13 years of longitudinal data, this study reveals the important multivariate trajectory across sleep quality, sleep duration, and cognitive function in China’s older adults. Based on the GBMT, we divided the multivariate trajectory of older adults into 4 latent groups: ‘Good-performance’ (51%), ‘Decreasing’ (26%), ‘Oversleep & cognitive impairment’ (12%), ‘Sleep-deprived’ (11%). Unlike a single trajectory analysis, based on 3 indicators (sleep quality, sleep duration, and cognitive function), each trajectory has a different combination, and more comprehensively reveals the aging changes of China’s older adults. This study further explores predictors of latent trajectory groups. We found differences between gender, age, and education across different latent trajectory groups. Finally, our study found that different latent trajectory groups are associated with all-cause mortality to varying degrees. Individuals in the ‘Oversleep & cognitive impairment’ had a 170% increased risk of all-cause mortality. The results of this study suggest that appropriate interventions can be designed to target the characteristics of trajectories, thereby reducing the risk of death. Remarkably, this is one of the few studies known to analyze the trajectories of simultaneous changes in sleep quality, sleep duration, and cognitive function, and examines the association between these changes and death.

In past studies, researchers have made unremitting efforts to investigate the association between sleep duration, sleep quality, and cognitive function, but the results have been inconsistent. On the one hand, researchers consider that sleep duration and sleep quality are beneficial predictors of cognitive function. On the contrary, a decrease in the quality and length of sleep may cause by cognitive decline. Due to the short observation time of previous studies, the real effect is often difficult to measure. Our 13-year follow-up study suggested that sleep disorder and cognition function appear to bidirectional association in late life. In our study, only participants in the ‘Oversleep & cognitive impairment’ group showed cognitive impairment and sleep duration and quality worsened, while another group of participants maintained good cognitive function despite declining sleep duration and quality.

On the one hand, the heterogeneity of these trajectories suggests that sleep worse is more likely to develop cognition decline. Although these mechanisms are unclear, there are several possible explanations. In past research, most studies tend to explain that disturbed sleep increases the risk of cognitive impairment. First, lack of sleep or poor sleep quality may affect the gene expression of circadian rhythms in specific areas of the brain (such as the thalamus, and hypothalamus), leading to brain dysfunction and subsequent cognitive impairment [29, 30]. Second, excessive sleep duration may lead to disturbances in circadian rhythms, which in turn lead to sleep disturbance and cognitive impairment [31]. To contrast, cognitive impairment may also lead to sleep disturbance. 70–80% of individuals with cognitive impairment suffer from sleep disruption [32, 33]. Individuals with cognitive impairment were more likely to report a higher number of nocturnal awakenings and more fragmented nocturnal sleep, resulting in poorer sleep quality [34]. Moreover, as individuals in ‘Good-performance’ group were younger and had good cognition function at baseline, we cannot rule out the possibility of cognitive impairment causing sleep problems. Our results suggest that there may be interdependence between sleep and cognition. Possibly due to the short follow-up, older adults with higher cognitive scores at baseline were not enough to observe cognitive decline even if they experienced sleep disorders. This suggests that sleep quality and sleep duration are modifiable predictors of early cognitive impairment. Our study cannot rule out bias by sampling.

Since 90% of the individuals were with lower education, caution is required in interpreting the internal relationships of these trajectories. Although it is commonly accepted that the association between education and cognitive function is positive, this association is not necessarily causal [35]. Past studies have shown that delayed cognitive decline is due to cognitive reserve about health knowledge instead of education [36]. In our study, although younger generations were more likely to have higher education, the latent trajectory remained an independent contributor to death after age grouping. Therefore, health literacy must be promoted among older adults with different levels of education to delay cognitive decline and even reduce the risk of death.

Our findings suggest that participants with different trajectory characteristics have varying degrees of association with death. This is consistent with previous studies. Previous studies have shown that excessive sleep duration and poor sleep quality increase the risk of death by 12–56% [37, 38], while cognitive impairment (score in the 25% quartile) increases death by 32% [39]. Our results showed that cognitive impairment accompanied by sleep problems had the highest risk of death, with a 170% increase. While participants in ‘Decreasing’ group, without cognitive impairment but with sleep problems, had a 51% increased risk of death. Our results suggest that individuals with cognitive impairment accompanied by sleep disorders may need to be prioritized when allocating limited public health resources.

Results of subgroup analyses by sex indicated that women had a higher mortality risk than men with the same trajectory characteristics. Although previous studies have shown that women’s objective sleep quality may be higher than men’s, women’s subjective sleep disruption such as more nighttime awakenings, difficulty falling asleep, and length of nighttime wakefulness, etc [40, 41]. Additionally, sleep problems may be due to differences in sex hormones [42]. Giving women estrogen therapy can reduce sleep disturbance, research has found. Therefore, when formulating sleep intervention-based strategies to prevent death, it is necessary to focus on sleep problems in women.

This study also has some limitations. First, sleep duration and sleep quality are self-reported, which may introduce misclassification bias. However, studies have shown that self-reported sleep duration correlates strongly with instrumentally measured, relatively objective measures of sleep [43, 44]. Second, although our study took some confounding variables into account as much as possible (such as physical activity, daily exercise, number of chronic diseases, etc.), it is still possible that residual confounding caused by measured or unmeasured variables (such as exercise intensity) cannot be ruled out Impact. However, the results of the subgroup analysis indicated that our findings were relatively robust. Third, the sample of this study can be representative of older Chinese aged over 60 years, so consistency with other age groups, regions, and ethnicities needs to be considered when generalizing the findings. However, there is currently no evidence that the extrapolation of the findings is unreasonable.

## Conclusions

In summary, through the national representative data followed for 13 years, we used the GBMT to identify 5 groups of trajectory based on sleep duration, sleep quality, and cognitive function. These results suggest that sleep and cognition are more likely to be complex two-way relationships. Further. We observed that these trajectory groups were associated with all-cause mortality, in which individuals with progressive cognitive decline and longer sleep duration, and poorer sleep quality had the highest risk of death. Intervention policies for cognitive impairment should emphasize the importance of sleep duration and quality, with a particular focus on individuals with low cognitive scores and poor sleep quality and duration. This helps reduce the personal and social burden of cognitive impairment in older adults, which in turn reduces the risk of death. Future studies are needed to further confirm the underlying association between sleep and cognition and to further confirm the impact of these changes on mortality.

### Supplementary Information

**Additional file 1.**

## Data Availability

The analytical dataset used in this study is a publicly available dataset released by the CLHLS. Information about the data source and available data are found at https://www.icpsr.umich.edu/icpsrweb/DSDR/studies/36179. Researchers can obtain these data after submitting a data use agreement to the CLHLS team.
