# Supplementary material for: Association between all-cause mortality and trajectories across quality and duration of sleep and cognitive function: based on Group-Based Multivariate Trajectory modeling
Source: BMC Geriatr. 2023 Aug 30;23:528. doi: 10.1186/s12877-023-04231-3 (PMC10468863; doi:10.1186/s12877-023-04231-3)
Supplement: Supplementary file 1 — Additional file 1. [file 12877_2023_4231_MOESM1_ESM.docx]

**Association between all-cause mortality and trajectories across quality and duration of sleep and cognitive function: based on** **Group-Based Multivariate Trajectory modeling**

Jianlin Lin, MM ^a,b,c,d^, Jian Xiao, MM^e^ , Qiao Li, MM^a,^ **^†^** , Li Cao, PhD ^a,^**^†^**

**Author Affiliations**:

1. International School of Public Health and One Health, Hainan Medical University, Haikou, China
2. Department of Oral and Maxillofacial Surgery, Stomatological Center, Peking University Shenzhen Hospital
3. Guangdong Provincial High-level Clinical Key Specialty
4. Guangdong Province Engineering Research Center of Oral Disease Diagnosis and Treatment
5. Nanjing Municipal Center for Disease Control and Prevention, Nanjing 210003, China

†. These authors contributed equally to this work.

**Corresponding Author**:

Qiao Li, MM, associate professor,

International School of Public Health and One Health, Hainan Medical University,

Add: No. 3 Xueyuan Road, Longhua District, Haikou 571199, Hainan, China,

Tel: +86-0898-66890169

E-mail: [hy0208011@hainmc.edu.cn](mailto:hy0208011@hainmc.edu.cn);

Li Cao, PhD, Professor

International School of Public Health and One Health, Hainan Medical University,

Add: No. 3 Xueyuan Road, Longhua District, Haikou 571199, Hainan, China,

Tel: +86-0898-66890169

E-mail: [hy0208037@hainmc.edu.cn](mailto:hy0208037@hainmc.edu.cn).

**Calculation of sample size**

In order to ensure the reliability of the study population and obtain sufficient samples, we selected CLHLS with national representation (CLHLS).The CLHLS is the largest and nationally representative survey of older adults in China, covering 22 cities and counties in 31 provinces of China. In general, age, urban-rural, and sex were taken into account and weighted in the calculation of sample sizes. The specific sampling method is as follows ([obtained from live.com](https://view.officeapps.live.com/op/view.aspx?src=http%3A%2F%2Fchads.nsd.pku.edu.cn%2Fdocs%2F20200408162356858614.doc&wdOrigin=BROWSELINK)).

For each centenarian, one nearby octogenarian (aged 80-89) and one nearby nonagenarian (aged 90-99) with pre-designed age and sex were matched and interviewed. We tried to have approximately equal numbers of male and female octogenarians and nonagenarians at each age from 80 to 99. We did not follow the procedure of proportional sampling, in order to avoid the errors of random fluctuation due to too small sample size at more advanced ages, especially for males. Consequently, appropriate weights should be used to compute the overall average for the oldest old persons age 80+ and the averages of the age groups (e.g. 80-89 and 90-99).

The age (x), sex (s), and rural-urban residence (r) specific weight w(x,s,r) is computed as:

w(x,s,r) = [ N(x,s,r) / ∑_x_∑_s_∑_r_ N(x,s,r) ] / [ n(x,s,r) / ∑_x_∑_s_∑_r_ n(x,s,r) ]

= [N(x,s,r) / n(x,s,r) ] * [∑_x_∑_s_∑_r_ n(x,s,r) / ∑_x_∑_s_∑_r_ N(x,s,r) ]

N(x,s,r) is number of persons of age x, sex s, and residence r, derived from projected 1998 oldest old population based on the 1990 census 100% data tabulations for the 22 provinces where the 1998 survey was conducted, and the estimated age-sex-specific survival probabilities between 1990 and 1998. We have no age-specific data about rural-urban migration between 1990 and 1998. We, therefore, used the overall proportion of urban population age 80+ (0.378) derived from our 1998 survey, which is the same as our expert estimate of the urbanization level in the 22 provinces in 1998, to adjust the projected 1998 rural-urban distribution. The n(x,s,r) is number of persons of age x, sex s, and residence r, derived from the 1998 healthy longevity survey. The weight is actually the multiplication of the ratio of [N(x,s,r) / n(x,s,r) ] and the factor [∑_x_∑_s_∑_r_ n(x,s,r) / ∑_x_∑_s_∑_r_ N(x,s,r) ]. The factor is the overall sampling ratio. No weights are needed when we compute the average of the centenarians age 100-105, since the survey attempted to interview all centenarians in the sampled areas. We did not include the very few cases of super-centenarians of age 106 and above due to possibility of questionable quality of their age reporting.

The weight w(x,s,r) is actually the ratio of age distribution of the entire population age 80+ in 1998 to the age distribution of the 1998 sample. The weights for the over-sampled extremely old persons (e.g. 90+) are less than 1.0, and weights for under-sampled elders (e.g. age 80-85) are greater than 1.0.

The values of the weights vary (usually greater than 1.0 under age 88 and less than 1.0 above age 90), and it produces correct proportions of certain attributes within age groups by using the weights. However, SPSS (or other software) would not produce correct p-values for testing the statistical significance of the differences of the proportions among different age groups, since the sub-sample size of the age groups are altered after weighting the individual cases. Therefore, the weights need to be adjusted to make sure that the sub-sample size within each age group after weighting is exactly the same as the true sub-sample size. Denote C_j_(s,r) as the adjusting factor for age group j (e.g. age group 90-95) with sex s and residence r; T_j_(s,r) as the total number of interviewed persons of the age group j with sex s and residence r. The following equations must be fulfilled: C_j_(s,r)*∑_x_w(x,s,r) n(x,s,r) = T_j_ (s,r). Solving this equation, we obtain the adjusting factor,

C_j_ (s,r) = T_j_ (s,r) / ∑_x_ [w(x,s,r) n(x,s,r)].

“∑_x_” here refers to the sum of the number of the persons over the age group (e.g. age group 90-95).

The adjusted weights are: w’(x,s,r) = w(x,s,r) * C_j_ (s,r). We should use the adjusted weights that produce both correct proportions, and correct sub-sample sizes and thus correct p-values for testing statistical significance of the differences of the proportions among various age groups.

If one computes proportions of certain attributes of age groups with rural and urban combined, the adjusting factor is not rural-urban specific, but age group (j) and sex specific:

C_j_ (s) = T_j_ (s) / ∑_x_ ∑_r_ [w(x,s,r) n(x,s,r)].

If one computes proportions of certain attributes of age groups with both rural and urban combined and sexes combined, the adjusting factor is neither rural-urban specific, nor sex-specific, but only age group (j) specific:

C_j_ = T_j_ / ∑_x_ ∑_r_  ∑_s_ [w(x,s,r) n(x,s,r)].

**Measures**

Regular exercise was measured by the question ‘Do you do exercises regularly at present’(no, yes). Smoke was evaluated by the question ‘Do you smoke at present’ (no, yes). The drink was measured by the question ‘Do you drink alcohol at present’(no, yes). The number of chronic diseases were measured by 21 items diagnosed by hospital, including heart disease, stroke/cerebrovascular disease, bronchitis/emphysema/asthma/ pneumonia, pulmonary tuberculosis, cataracts, glaucoma, cancer, prostatic diseases, gastric/duodenal ulcer, Parkinson’s disease, bedsore, arthritis, epilepsy, cholecysitis/cholelith disease, blood disease, rheumatism/rheumatoid disease, chronic nephritis, galactophore disease, myoma of uterus, hyperplasia of prostate, and hepatitis. The number of chronic was categorized as 0, 1-2, and >2.

References

1. Banister, J. 1990. Implications of aging of China's population. In: Zeng Yi, Zhang Chunyuan and Peng Songjian (eds.), Changing Family Structure and Population Aging in China: A Comparative Approach. Peking University Press, 1990. Beijing. And in: Dudley L. Poston, Jr. and David Yaukey (eds.), The Population of Modern China, New York: Plenum Press, 1992.

2. Christensen K. and J.W. Vaupel. 1996. Determinants of longevity: genetic, environmental and medical factors. Journal of Internal Medicine. 240.

3. Coale, Ansley and Shaomin Li. 1991. The effect of age misreporting in China on the calculation of mortality rates at very high ages. Demography. Vol. 28, No. 2.

4. Colvez, A., and J.M. Robine (1986): L’Esperance de Vie Sans Incapacite en France en 1982, Population, 41 (6): pp. 1025- 1042.

5. Crimmins, E.M., M.D. Hayward, and Y. Saito (1994): Changing mortality and morbidity rates and the health status and life expectancy of the older population, Demography, 31, pp. 159- 175.

6. Crimmins, E.M., M.D. Hayward, and Y. Saito (1996): Differentials in active life expectancy in the older population of the United States, Journal of Gerontology: Social Sciences, 51B (3), pp. S111-S120.

7. Crimmins, E.M., Y. Saito, and D. Ingegneri (1989): Changes in life expectancy and disability-free life expectancy in the United States. Population and Development Review, 15 (2): pp. 235-267.

8. Fillenbaum, G.G. 1988. Multidimensional Functional Assessment of Older Adults.

9. Finch Caleb E. 1990. Longevity, Senescence and Genome. Chicago. The University of Chicago Press.

10. Jeune B. 1995. In search of the first centenarians. In: Jeune B. and J. Vaupel (eds.). Exceptional Longevity: From Prehistory to the Present. Odense University Press, pp. 11 24.

11. Ju, Chen Ai and Gavin Jones. 1989. Aging in ASEAN: its socioeconomic consequences. Pasir Panjiang, Singapore: Institute of Southeast Asian Studies.

12. Lamb Vicki L. 1999. Active life expectancy of the elderly in selected Asian countries. NUPRI Research Paper Series No. 69. Nihon University, Population Research Institute. Tokyo, Japan.

13. Manton, Kenneth G. and Kenneth C. Land. 1999. Active life expectancy estimates for the U.S. elderly population: A multidimensional continuous mixture model of functional change applied to complete cohorts, 1982-1996. Paper presented at PAA, New York.

14. Ogawa, Naohiro. 1988. Aging in China: demographic alternatives. Asian Pacific Population Journal, No. 1.

15. Penning, Margaret J., Laurel A. Strain. 1994. Gender differences in disability, assistance and subjective well-being in later life, Journal of Gerontology: Social Science, 49: s204.

16. Pressley, Joyce Carolyn. 1996. Factors associated with change in functional state and health care utilization in the elderly. UMI Dissertation.

17. Robine, J. M., C. D. Mathers, D. Bucquet (1993). Distinguishing health expectancies and health-adjusted life expectancies from quality-adjusted life years. American Journal of Public Health. 83(6): 797-8.

18. Rogers, A., R. Rogers, and L.G. Branch (1989): A Multistate Analysis of Active Life Expectancy, Public Health Reports, 104: 222-226.

19. Rogers, A., R. Rogers, and L.G. Branch (1990): Longer life but worse health? Measurement and Dynamics, The Gerontologist, 30: 640-649.

**Supplemental Table 1 Distribution of education levels by age groups**

| **Age group** | **Illiterate** | **Literate or primary school** | **Junior high and above** | **P-value*** |
| --- | --- | --- | --- | --- |
|  | **N(%)** | **N(%)** | **N(%)** |  |
| **65-74 years** | 84 (6) | 1066 (67) | 433 (27) | <0.001 |
| **75+ years** | 94 (9) | 732 (71) | 212 (20) |  |

* P-values were obtained from the Chi-square test.

**Supplemental Table 2 The association between the latent trajectories and all-cause mortality grouped by age based on Cox regression.**

|  |  | **Younger (<75)** | **Older (≥75)** |
| --- | --- | --- | --- |
| **Group** | Good-performance |  |  |
|  | Decreasing | 1.63 (1.14-2.32, p=.007) | 1.57 (1.26-1.95, p<.001) |
|  | Oversleep & cognitive impairment | 2.43 (1.28-4.59, p=.006) | 3.53 (2.75-4.53, p<.001) |
|  | Sleep-deprived | 1.11 (0.79-1.55, p=.544) | 1.13 (0.81-1.58, p=.474) |
| **Gender** | Women |  |  |
|  | Men | 2.67 (1.90-3.75, p<.001) | 1.47 (1.16-1.87, p=.002) |
| **Education** | Illiterate |  |  |
|  | Literate or primary school | 0.84 (0.53-1.34, p=.466) | 1.06 (0.78-1.44, p=.696) |
|  | Junior high or above | 0.48 (0.28-0.84, p=.010) | 0.74 (0.51-1.09, p=.132) |
| **Occupation** | Low level |  |  |
|  | High level | 1.14 (0.79-1.64, p=.490) | 1.04 (0.81-1.33, p=.768) |
| **Residence area** | Rural |  |  |
|  | City | 0.97 (0.74-1.26, p=.807) | 0.83 (0.68-1.01, p=.061) |
| **Current marital status** | No |  |  |
|  | Yes | 0.76 (0.57-1.02, p=.069) | 0.77 (0.64-0.93, p=.005) |
| **Regular exercise** | No |  |  |
|  | Yes | 0.92 (0.71-1.19, p=.513) | 0.86 (0.71-1.04, p=.112) |
| **Drinking** | No |  |  |
|  | Yes | 0.88 (0.68-1.14, p=.330) | 0.82 (0.67-1.01, p=.059) |
| **Smoking** | No |  |  |
|  | Yes | 0.92 (0.71-1.19, p=.535) | 0.96 (0.78-1.18, p=.682) |
| **Disability** | No |  |  |
|  | Yes | 1.13 (0.45-2.88, p=.790) | 1.10 (0.79-1.53, p=.571) |
| **Number of chronic** | 1 |  |  |
|  | 1-2 | 1.27 (0.96-1.67, p=.096) | 1.11 (0.90-1.35, p=.330) |
|  | >2 | 1.02 (0.64-1.63, p=.925) | 0.94 (0.69-1.29, p=.714) |
